# “Scie-losophy” a teaching and learning framework for the reconciliation of the P4C and the scientific method

**DOI:** 10.1016/j.mex.2023.102417

**Published:** 2023-10-05

**Authors:** Amal A. Alsufyani

**Affiliations:** a College of Science and Health Professions, King Saud bin Abdulaziz University for Health Sciences, Jeddah, Saudi Arabia; b King Abdullah International Medical Research Center, Jeddah, Saudi Arabia; c Ministry of the National Guard - Health Affairs, Jeddah, Saudi Arabia; d Montgomery College, Rockville, MD, United States

**Keywords:** Scientific method, Philosophical education, P4C, Inquiry, Science education, Education, Problem-solving, Philosophy, STEM, “Scie-losophy” a framework for developing metacognition and intelligence

## Abstract

Today's students face new challenges that demand high levels of intelligence and meta-thinking skills. Science-based educational pedagogies like STEM, 5E's, and discovery-based education have earned a strong reputation for nurturing children's reasoning and critical thinking skills. However, there's a need for them to be more open and conceptual in order to better prepare students for modern life's challenges. The realms of philosophical and scientific-based educational models currently dominate the educational arena. In retrospect, both models have inherent values that would benefit learners. It is beneficial to examine both methods to develop a teaching framework that fosters higher-order thinking, metacognition, and problem-solving skills. The objective of this paper is to delve into the essenceof the philosophy for children (P4C) method in comparison with the scientific method and their impact onstudents' learning. Using the six reasoning strands, I will systematically compare the two models for strengths and similarities. Throughout this comparison, I aim to maintain objectivity by by drawing on references and practical experience, avoiding any undue bias in favor of one model over the other. Subsequently, by applyingTrompenaars Hampden-Turner™ dilemmas reconciliation model, I will propose the Scie-losophy model, which represents reconciliation between the two methods for the benefit of the learner and the greater good of society.

• Currently, philosophical, and scientific-based education models dominate the educational arena. Both models have inherent values that would benefit learners.
• Adopting one approach above the other will yield less than optimal results. I strongly discourage following a saturated educational system in which one of the two methods is used exclusively.
• I propose a model that represents reconciliation between the two methods for the benefit of the learner and the interest of society.

## Graphical abstract

The reconciliation of the P4C versus the scientific method dilemma.

**Figure:**
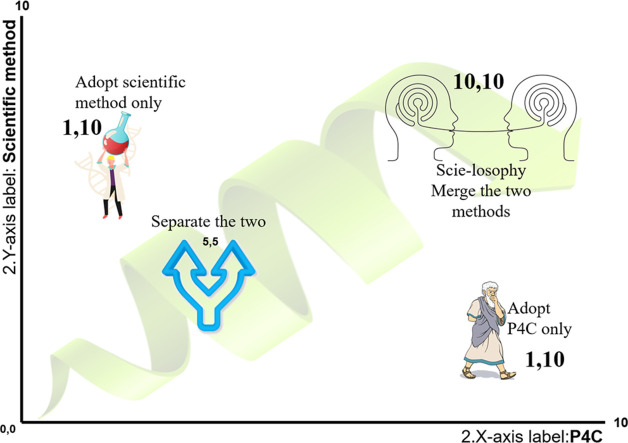

Specifications table

**Table:** 

Subject area:	Biochemistry, Genetics and Molecular Biology
More specific subject area:	*Science Education*
Name of your method:	“Scie-losophy” a framework for developing metacognition and intelligence
Name and reference of the original method:	*The Scientific method* Fowler, S., Roush, R., & Wise, J. (2013). *Concepts of biology*. OpenStax College, Rice University
Resource availability:	*Varies depending on the topic being studied or learned*

## Method details

While training to become an accredited P4C facilitator, I grew interested in the distinction between philosophy for children (P4C) and scientific methodology. P4C is an attempt to systematically reconstruct learning techniques for children; it is a student inquiry-based system that centers on creating spaces for learners to question, reason, and explore ideas. On the other hand, the scientific method is a systematic practical-logical framework to inductively and deductively reason through observation to form and test hypotheses. Even though the scientific method is a process applied by scientists to make scientific discoveries, it is being used as a method of teaching and a way of thinking and learning in science classrooms to logically write conclusions. The supporters of P4C call for allowing students to think for themselves using a claim similar to this used by the advocate of the experimental-based scientific method: (The only knowledge one really possesses and understands is the knowledge that one discovers by oneself [1]). How does P4C stand out in comparison with other communities of inquiry? Furthermore, Will adopting the P4C methodology be sufficient on its own? Is it sustainable? I asked my instructor, “What makes P4C distinguished?” and they answered, “The community of inquiry”. However, scientists are also a community of inquiry.

The concept of 'Community of Inquiry' was first introduced by philosopher, mathematician, and scientist Charles Peirce and philosopher and psychologist John Dewey. It concerns the nature of knowledge formation and the process of scientific inquiry. The Community of Inquiry framework calls for creating deep and meaningful learning experiences by focusing on three interdependent elements: social, cognitive, and teaching presence. It was clear to me that there is an overlap between the scientific methodology and P4C, but I was concerned by the rigidity of some scientific methodologies and the practice of P4C as a standalone method of teaching. I wanted to determine how each of the two fields if they must be separate, can complement and benefit the other. Moreover, in this study, we will refrain from using the term community of inquiry (COI) to refer to P4C, as seen in many pieces of literature.

The article, “The Essential Six Strands,” [2] mentions six strands that characterize best practices in teaching. The six strands, which I will apply to evaluate both P4C and the scientific method, are **inquiry**-inspired, **concept**-constructing, dialog-driven, **reason**-respecting, **reflection**-reliant, and **virtues**-valuing. These six strands are the proposed metrics to achieve good teaching. Sutcliffe has acknowledged that “these six strands can be aligned closely with only two or three strands or emphases in the International Baccalaureate (IB) curriculum,” [2] which I will challenge below.

Sutcliffe further stated, “I still believe that the framework I am offering is more comprehensive, more coherent, and yet more straightforward than any other” [2]. As a staunch believer in the overlap and exchange of knowledge, I am concerned that the complete isolation between the scientific method and P4C and perceiving them as competitors will be disadvantageous.

### What is P4C?

According to SAPERE, “P4C is an approach to teaching and learning that explores the big ideas that arise in all areas of education and life experiences. P4C uses philosophical dialog and inquiry to help learners think, speak, listen, learn, and live together more effectively” [3].

In P4C, there are ten steps (Table 1) that offer a process for thinking about a stimulus. The first of the ten steps ensures that students are prepared to receive a stimulus. Other steps include proper presentation of a stimulus, adequate thinking time, a systematic way of selecting a question to be answered, and a flow of discussion that supports reflection [2]. Thus, P4C is a student-centered methodology that is process-driven. Presenting P4C in comparison to other methods implies an invitation to differentiate itself in the realm of education and create a distinction where P4C is the superior teaching method. Some advocates of P4C propose replacing the other educational systems, such as IB, and promoting P4C as the best way forward. However, is this what the vision of P4C truly claims? Was this what Matthew Lipman intended, when he stated, “I am now convinced that philosophy can and should be a part of the entire length of a child's education” [4]? Will P4C be able to replace other pedagogies?

**Table 1:** Summary of the original ten steps of P4C [5].

Phase	Summary
Preparation	This is about getting the group into P4C 'mood/mode. In the early days, activities might be geared toward building a sense of community, but later they might focus more on the development of thinking and inquiry skills. Don't forget occasionally to rehearse the aims or guidelines of P4C.
Presentation (of stimulus)	The stimulus should be engaging, relevant, and meaningful to the group. It should contain some 'big' (i.e. Common, Central, and Contestable) ideas/concepts that will inspire philosophical questions.
Personal Response (Thinking Time)	Quite simply, time for private reflection on the stimulus. Silent thought can be challenging and may need to be modeled and developed over time. Encourage the use of paper to jot down ideas.
Question-making	Small group conversations. After sharing their personal responses to the stimulus, groups create open, discussible questions to put forward to the class. (Usually groups of 3 - 5, so as to end up with 6 - 10 questions).
Questions-airing	Questions, prominently displayed, perhaps on the floor, are celebrated, analyzed, and compared. Ambiguity or vagueness is cleared up, and links are often suggested, but also significant differences are noted.
Question-choosing	One question is agreed upon for the focus of the inquiry/dialog to follow. The question is either chosen by the community (usually by voting) or negotiated by the facilitator.
First words	Getting the inquiry/dialog started. One way is to invite the group whose question is voted for to explain their thoughts on it. Think-pair-share can be a good starter, too, or asking for a proposal/ response to the question. Over time, more critical responses can be encouraged, e.g. identifying assumptions in the question or writing to reply (first thoughts noted in writing).
Middle words	Once the question/dialog opens up, the metaphor of 'building is key: building on each other's ideas, and towards a better understanding of the concepts/issue(s) arising. It is good practice to pause midway for reflection on how the building is going. What progress has been made?
Last words	A chance for pupils to offer their final words on what has been discussed. Often those who haven't contributed during the session do so here and show they have been engaged. Different foci may be suggested.
Review	Basically, www.ebi (what went well; even better if) - best done after a short break (or at the end of the day). The most important is to use this step for the planning of the next session. Suggestions for lines of further inquiry and skills development can be invited and agreed upon. Invite stimuli suggestions.

Verbal and written discourse and discussion with P4C trainers and facilitators suggest that P4C is offered as a complement to other fields. However, in practice, P4C is presented as a separate standalone frame of teaching for all subjects. It's worth noting that “while philosophers do not agree among themselves on either the range of proper philosophical questions or the proper methods of answering them, they do agree that merely expressing one's personal opinions on controversial topics like these is not doing philosophy” [6]. Nonetheless, accredited P4C facilitators are often pereceived or presented as trained somehow in facilitating philosophical discussions. Therefore, the current application of P4C could be presented as simply discussion facilitation rather than a practical or applied philosophy session. What's more, with the sole incorporation of P4C, education might rely solely on theory and hypothesis. In some cases, albeit rare in classrooms, a discussion could logically lead to a conclusion that contradicts empirical evidence. Consequently, as someone knowledgeable in both philosophy and science, I was led to explore the strengths and weaknesses of the scientific and P4C methodologies, respectively.

### What is the scientific methodology?

**Fig. 1:**
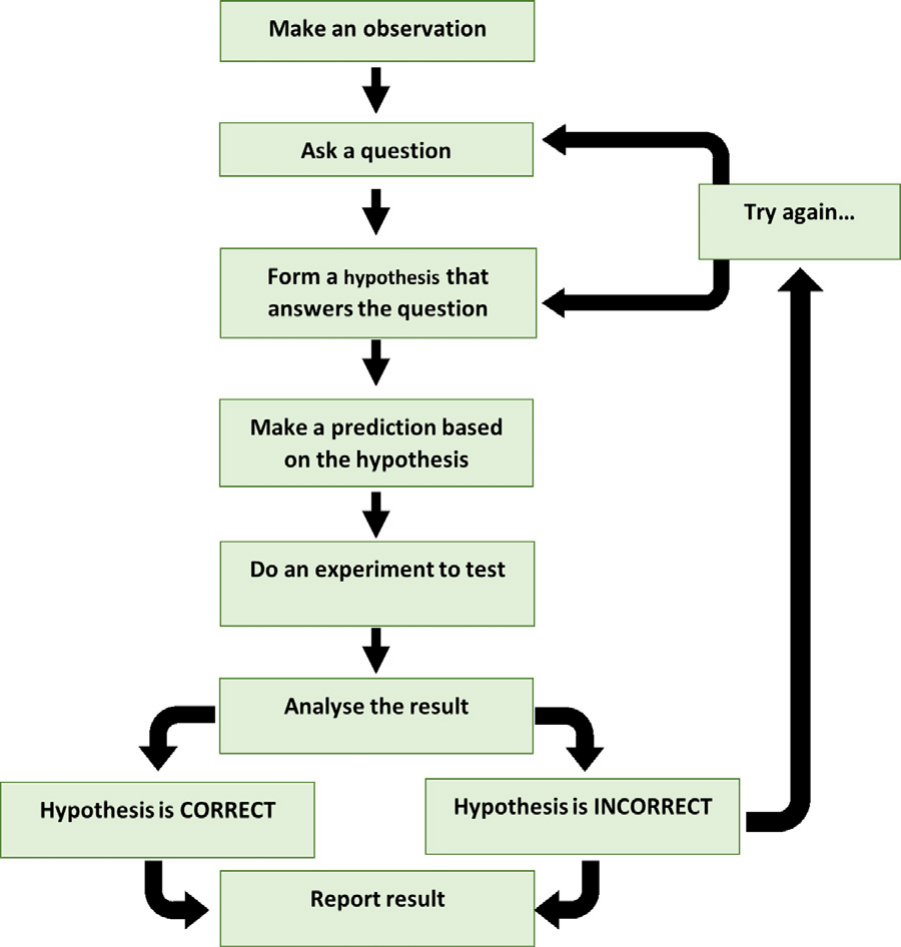
Steps of the Scientific Method [8].

The scientific methodology (Fig. 1) is a systematic process that starts with an observation and continues with the formulation of a question, construction of a hypothesis by answering the question, experimentation, inductive and deductive reasoning, acceptance or rejection of the hypothesis, and finally, making theories and conclusions [7].

In meetings and correspondence with Barbara Houtz, the CEO of STEM Education Solutions and an educational consultant at the National Institutes of Health, she described the evolution of learning strategies. She emphasized the limitations od certain strategies "that have been completely disproven by more rigorous methods, including learning styles theories, constructivism (which is only effective if you have inquisitive, participating, motivated students who have formed opinions about the natural world BEFORE entering a science classroom), and inquiry-based learning versus direct instruction. Educational research is still being challenged to be as rigorous as scientific research, but it's not there yet.” Furthermore, Houtz commented on her experience working with elementary and secondary school teachers teaching scientific methods who have not done the research themselves: “The scientific method taught by teachers untrained in science labs is mechanical. While applying the scientific method in educational settings, teachers follow the steps as a checklist. Teachers are observing students as they follow the steps of the method to reach the answer in the teachers' minds. In this way, there is no room for open-ended questions or exploration of different interpretations, and thus the scientific method approach is rigid.” Houtz's analogy described untrained teachers who are teaching the scientific methods as “cooks” whereas scientists who understand the inquiry essence of research are “like Chefs” who deeply understand how to create more questions out of the answers collected from the experiment.) The key takeaway is the importance of incorporating a freely structured method that allows open-ended inquiry, where teachers are not required to check the work of a student based on their ability to reach a correct answer in the book, but instead based on the ability to reason their answer and support it with evidence. An underlying hindrance to the transition to such an open-minded education is the fear of uncertainty, acceptance of mistakes, and admission of limited knowledge that the teachers must deal with. These insights from Barbara Houtz, shed light on the challenges and opportunities in education. Understanding these insights sets the stage for a deeper analysis of how pedagogical approaches can shape modern education.

Although the P4C and the scientific methodology are not the only two processes in education, these two models dominate the educational arena. Historically, philosophy and science have been very tightly linked; however, there has been a continuous battle between scientists and philosophers. Some scientists consider philosophy antagonistic [9] and unprogressive [10]. This appears in statements like the one made by the physicist Lawrence Krauss “Science progresses and philosophy doesn't” [11]. Philosophers, on the other hand, have opinions that are critical of scientists and the scientific method. James Blachowicz, author of the New York Times article “There Is No Scientific Method” said “the method on which science relies exists wherever we find systematic investigation,” and that systematic investigation such as quantified precision “is not to be confused with a superior method of thinking.” [12]. The epistemological argument between scientists and philosophers poses a danger in viewing them as competitors rather than collaborators in the design of our educational systems. This is apparent from the US education's reverence for the “laboratory” and the “scientific method” [1], but not the UK where we see an adoption of P4C. Understanding this historical context is crucial as we examine the philosophical and scientific underpinnings of various pedagogical approaches.

Some pedagogies incorporated the hands-on practical scientific method to be applied in school including the “Learning by discovery” [1]. An attempt by scientists to develop a more open-ended inquiry-based version of the scientific method can be seen in the 5E Instructional model, which was developed by the Biological Sciences Curriculum Study in 1987 (Table 2). At first glance, you can consider the 5E model as a fused version of both P4C and the scientific method. However, by examining and closely working with the scientific method and P4C, the 5E model lacks important steps that are explained in detail in the scientific method and P4C. Thus, I have decided in this article to consider the most recent full version of the scientific method and comment on the 5E model in the appropriate relative places.

**Table 2:** Summary of the BSCS 5E Instructional Model [13].

Phase	Summary
Engagement	The teacher or a curriculum task accesses the learners’ prior knowledge and helps them become engaged in a new concept through the use of short activities that promote curiosity and elicit prior knowledge. The activity should make connections between past and present learning experiences, expose prior conceptions, and organize students’ thinking toward the learning outcomes of current activities.
Exploration	Exploration experiences provide students with a common base of activities within which current concepts (i.e., misconceptions), processes, and skills are identified, and conceptual change is facilitated. Learners may complete lab activities that help them use prior knowledge to generate new ideas, explore questions and possibilities, and design and conduct a preliminary investigation.
Explanation	The explanation phase focuses students’ attention on a particular aspect of their engagement and exploration experiences and provides opportunities to demonstrate their conceptual understanding, process skills, or behaviors. This phase also provides opportunities for teachers to directly introduce a concept, process, or skill. Learners explain their understanding of the concept. An explanation from the teacher or the curriculum may guide them toward a deeper understanding, which is a critical part of this phase.
Elaboration	Teachers challenge and extend students’ conceptual understanding and skills. Through new experiences, the students develop deeper and broader understanding, more information, and adequate skills. Students apply their understanding of the concept by conducting additional activities.
Evaluation	The evaluation phase encourages students to assess their understanding and abilities and provides opportunities for teachers to evaluate student progress toward achieving educational objectives.

## Method

The study design included the following research objectives:

1. Evaluate the presence of the six essential strands of effective learning in the scientific method and P4C.
2. Use Trompenaars Hampden-Turner™ model to define the problem in terms of dilemmas, and codify these dilemmas in a standard style.
3. Propose a modified open-minded practical teaching framework.

### The six essential strands of effective learning

Roger Sutcliffe has proposed that there are six essential strands of effective learning. Ab Wahab (2022) emphasized the importance of paying attention to the lack of transparency, subjectivity, author bias, recruitment bias, and publication bias in studies that report on P4C advantages, and he concluded that there is a lack of systematic literature review which is needed for transparency and comprehensive structure [14]. In the scope of this article, we will systematically examine and compare the presence of the six strands of philosophical teaching most conducive to learning in both P4C and scientific methodologies. I will examine some strands, namely **inquiry**-inspired, **concept**-constructing, and **virtues**-valuing using the available literature because they are theory-based. On the other hand, I will comment on the remaining strands, dialog-driven, **reason**-respecting, and **reflection**-reliant, which are identifiable in practice based on my personal observations in P4C sessions.

We will test the presence of each strand in each of the two methodologies based on how scientists and philosophers view themselves. One must note that the scientific method is not equivalent to the aim and product of science, and P4C is not equivalent to the aim and product of philosophy. These two methods are the processes by which these targets are met [7].

The pressing questions are: Will promoting P4C as a standalone method in schools be beneficial? Can P4C alone foster logic and reasoning? If P4C is not sufficient alone, is that only because the current educational systems work to train students to meet certain career-targeted skills, and thus administrators must adhere to the currently approved educational system? Or because there are epistemological values to be missed if we solely adopt P4C?

### Trompenaars Hampden-Turner™ dilemma reconciliation model

**Fig. 2:**
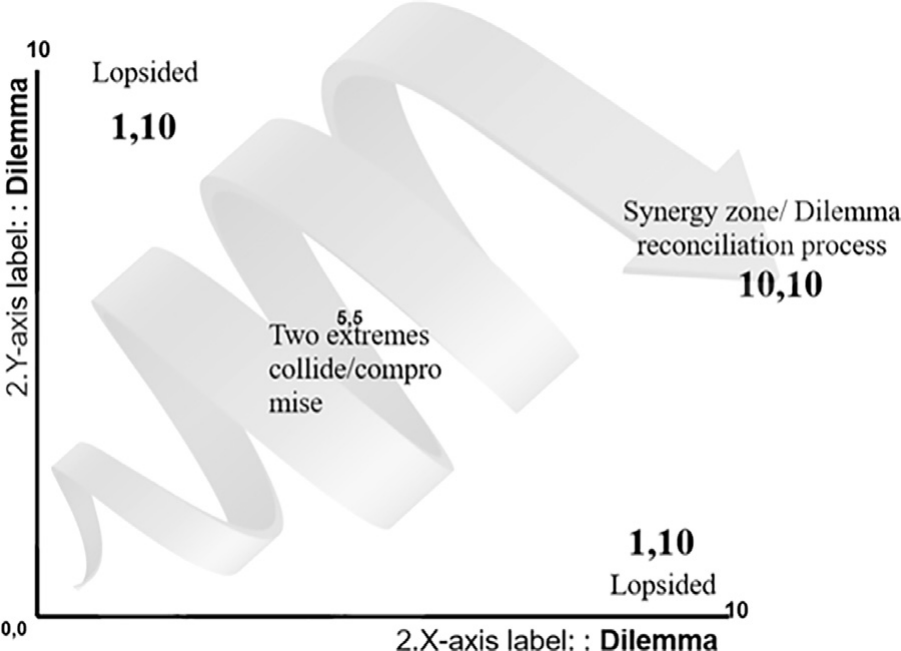
Dilemma reconciliation model [17].

Following the analysis, this article will use the dilemma reconciliation model proposed by Trompenaars Hampden-Turner to systematically [15] present a reconciliation between the Scientific, and Philosophy 4 Children methods and finally propose the modified Scie-losophy model of education. The dilemma reconciliation model (Fig. 2) is a practical model that was developed by Trompenaars Hampden-Turner™, in their consulting practice to put the dilemma theory in practice for leaders and managers when they are faced with a real word Dilemma [16]. This paper will work through the model of Trompenaars Hampden-Turner™ to define the problem in terms of dilemmas, codify these dilemmas in a standard style by placing the scientific method of teaching on one axis and the P4C on the other, and then provide the model (reconciliations) to these two teaching approaches until you reach the synergy zone (10,10).

### Modified teaching pedagogy

Based on the analysis in objectives one and two, a new framework for practical and open-minded pedagogy will be proposed.

## Results and discussion

### The six essential strands of effective learning

Examining the two methods using the six strands’ lenses revealed similarities and slight differences that are worth paying attention to. Each strand comparison is discussed below.

#### Inquiry-inspired

To begin, an inquiry is a group of activities to acquire and change beliefs about the world [18] The scientific community of inquiry defines scientific inquiry as the set of activities in which we study the world around us. This includes proposing explanations, gathering evidence and data, raising questions, and developing a plan for investigation and review [19].

Socrates has been associated with teaching throughout history, due to his dedication to education. In his community of inquiry, he placed great importance on students’ participation in education by inviting them to ask questions. He also valued the community as an integral part of building knowledge, where every member of the community becomes the seed upon which the dialog is built [20]. Socrates’ inquiry is characterized by questioning and investigation. One of the most important pillars of Socratic philosophy is to focus on motivating people to question. Questions are powerful tools to move from the comfortable state of knowing to the admittance of ignorance. Admitting ignorance creates the possibility of learning, which is the purpose of the Socratic method [21].

However, the traditional Socratic method has been criticized for going beyond an interrogative style, to the point of becoming argumentative, with a student-teacher-centered class [22]. In a P4C discussion, the Socratic model is adopted with modifications. There is a distinction between the type of questions in P4C and the questions in the Socratic method. P4C can be described as a gentler version of Socratic inquiry [23]. P4C softens the Socratic method by applying skills of community-based learning, deep listening, and a genuine search for wisdom.

In science classrooms, there are four types of inquiry: open, guided, coupled, and structured [19]. Open scientific inquiry is the student-centered and student-driven approach [24], which is closely comparable to P4C inquiry. In this method, the stimulus is presented by the teacher, and the students are asked to formulate questions based on their observations, predictions, and analysis. However, it is typically preferred in a science classroom, that students have prior knowledge about the topic [25]. Like how P4C starts by presenting a stimulus, the first step of the scientific method is to make an observation, which also implies a stimulus being observed. In P4C, the presentation of a stimulus is the second step because facilitators consider the opening exercise as an individual step in the process whereas scientists regard the opening as a teaching skill, but not part of the scientific methodology. In other words, scientists may use the P4C method of the presentation of a stimulus, even though the class will start with an alternate opening exercise.

Both methods rely on presenting the students with a stimulus. The scientific method puts an emphasis on observation, which includes gathering evidence from the environment or a stimulus that triggers the students [25]. P4C gives students a pause to think after the stimulus presentation, which is akin to observation. In light of these facts, both methods rely on making observations on a stimulus, thinking deeply about these observations, and questioning them.

Questions are a great tool to leverage observations and to produce knowledge related to the stimulus. Philosophers argue that the intention and function of the question are what makes philosophy, rather than the wording. Philosophy involves deep, complex thinking to answer questions. Thus, both open and closed-ended questions can be philosophical. A closed-ended question can be philosophical if it evokes thinking about the deep issues associated with it in order to answer it. On the other hand, the function of a question is philosophical if it involves solving a puzzling problem, solving a wonder, and cognitive dissonance and incongruity [26].

P4C puts great value on questioning. As seen in Table 1, steps four through six deal with question formation and selection. Since P4C is meant to teach students to think philosophically, it is fair to assume that the goal of questions in P4C is to be philosophical. Yet, no conditions or restrictions are put on questions selected during a P4C discussion. There are questions that involve question analysis. However, there are no clear definitions of which questions are to be asked or selected. Teachers are prohibited from working with students in order to enhance the quality of a question. The training of P4C facilitators has been slightly modified by several institutes like SEPARE, the Philosophy Foundation, Dialogue Work, and the Philosophy Man, to incorporate thinking tools from other pedagogies. Table 3 shows the modified model [27] that we practically use in discussion facilitation with Step four (of thinking and talk time) incorporated from training utilizing a shorter P4C model [28]. Some of these added tools are redirected from being used to help teachers ask better questions and instead used by facilitators with students, so the students gradually develop an awareness of the quality of their questions.

**Table 3:** Modified basic structure of P4C session (modified P4C) [13,27,28].

Step	Explanation
Step one: Circle	Sit in a circle so everyone can see each other. In online sessions, we encourage camera opening.
Step two: Community	Build a community by selecting the correct opening exercise.
Step three: Stimulus	Share a stimulus
Step four: Thinking-talk time	Allow a thinking time and/or talking time about the stimulus.
Step five: Concept extraction	Identify the concepts and main ideas raised by the stimulus
Step six: Question making	Individuals, pairs, or group thinking and discussion time to make questions follow
Step seven: Question analysis and selection	Work on questions by linking them to concepts and identify logic and hypothesis behind them. Then using any voting techniques, allow the group to vote on a single question.
Step eight: Discussion	Start a discussion about the question. The group that selects the question has the right to start the discussion by an attempt to answer the question. Then the discussion flows.
Step nine: Summary	At the end a final word can be given by any participant. It is encouraged to invite a member who did not share a lot of thought to say the final word that summarizes the discussion.
Step ten: Reflection	At the end participants reflect on the quality of the discussion: what went well? What can be improved?

Asking questions is an integrated part of the scientific method as well. To be able to formulate a scientific question is at the core of science [29]. Scientific questions meet the philosophical questions in their function to solve a problem raised from puzzling observations that cause cognitive dissonance [24]. Scientific questions come in many forms; they can be theoretical, explanation-based, postulation-based, evaluation of evidence, justification reasoning, or clarification of doubts [30]. Scientific research questions stimulate scientific inquiry. Thus, formulating a good scientific research question is crucial for designing the following process of experiments or theoretical analysis.

When students ask scientific questions, it reflects a lack of knowledge on their part [30]. But isn't admitting the lack of knowledge one hallmark of Socratic philosophy? In science, the cognitive processes associated with navigating a puzzling problem allow the formulation of the problem in a way that can be analyzed and studied [24]. However, in reality, students are rarely allowed to ask their own scientific questions in a classroom environment. One can say ideally that P4C sessions may supplement conventional teaching by creating an environment that welcomes questions.

Up until this point, it is clear that both the P4C and scientific methodology stimulate a community of inquiry. The focus is on value observation and thinking about these observations, but also on allowing students to formulate questions themselves. However, P4C reserves a clear space for students to formulate questions. In the majority of cases I have witnessed, although students are provided with the freedom to question, there are certain situations in which the facilitator provided the questions to be discussed. On the other hand, the scientific method does a better job of placing value on preparation and gathering information about the problem.

#### Concept-constructing

The human mind has an amazing capacity for abstraction, a great tool to extract important context out of distractors [31]. It is safe to assume that conceptual knowledge can be found in much of the sensory information surrounding us. Even the simplest tangible things, such as water [32,33], contain abstract information. Thus, abstraction is the essence of concept construction [33].

Concept-extraction is embedded within the traditional ten steps of P4C (Table 1) despite not having its own step. The learners presumably work with concept extraction in order to engage in effective discussion. A good stimulus should contain concepts that stimulate philosophical discussion, which in this sense does not offer much more than the scientific method. In the modified ten steps (Table 3), concept extraction is an integrated part. The fifth step is reserved for concept extraction and the sixth step is where participants link concepts to the formulated questions. This is a notable modification to the original P4C ten steps, which I believe can be integrated into my proposed methodology. Implementation can occur in many fields such as computer sciences, artificial intelligence, and physics because these fields often involve abstraction even though they are scientific fields [31,34].

However, it would be interesting to study the product of integrating concept extraction into the scientific method to see what hypotheses students may come up with when abstracting a scientific concept. It is of great importance to note that this method of concept extraction is the use of abstraction to enhance deductive reasoning, which is prominent in many fields such as mathematics.

Once abstracted, these concepts can be built upon. Constructivism is an important theory in education that states that people actively construct information instead of receiving knowledge [35], [36], [37]. An important outcome of both P4C and the scientific method is the construction of concepts. P4C claims that students construct knowledge during discussions to answer questions. However, it is key to define what constructivist theorists are referring to here.

There has been a lot of discussion about constructivism in P4C [38] and scientific education [39], [40], [41], [42]. In his research, Golding explored what kind of philosophical knowledge children gain from practicing P4C. He emphasized the necessity to distance P4C from the extreme Socratic constructivism approach [38]. Without digging deeper into constructivism definitions from both schools, for the purpose of this review, constructivism will be defined as the practice of building on well-curated knowledge. By this definition, both P4C and the scientific method satisfy the concept constructing criteria. With that said, constructivism in the scientific method is supported by a stronger body of knowledge.

#### Dialog-driven

It is apparent that P4C practices dialog through the use of discussion among group members, particularly during discussion time (Tables 1 and 3). Martin has discussed in his paper “The Philosophy of Logical Practice” the social aspect of the scientific and mathematical community of inquiries. He suggested that the scientific and mathematical communities build their own knowledge by means of a series of logical knowledge agreed upon in expert circles [43]. During the scientific method, students are urged to discuss with their peers and present their data. In many versions of the scientific method, a final step is clearly visible, asking researchers to discuss and share their findings. Although both methods have a dialog component in terms of communicating and discussing knowledge, I believe the P4C method is focused more on satisfying the dialog-driven component. This is a significant component that can enrich the learning process and should be considered in the modified version of the scientific method.

#### Reason-respecting

In P4C, students are expected to reason while formulating their argument or when they take a position, and they are asked to verbalize why they have chosen a certain position. When looking at the steps of the scientific method (Fig. 1), you can clearly see that reasoning is integrated. Students are provided a system by which they test their hypotheses, which are to be falsifiable and testable. Then based on the results that answer their scientific question, they either reject or accept the hypothesis. If the hypothesis is rejected, then the prediction should be examined, and another experiment should be done. Both results can add more observations, and new questions may be asked. I am strongly convinced that the scientific method does a better job of providing a framework of practical-based reasoning that can be adapted by P4C to enhance the quality of philosophical discussion.

#### Reflection-reliant

In the scientific method, reflection is vital. However, it is normally expressed in writing as implied in the dialog-driven discussion above. Students write their reflections on the process, problems they may confront throughout their research, and predictions for the outcome. In the original models of P4C, (Table 1) reflection comes in the review step. In comparison, the modified model (Table 3) does a better job of presenting the reflection. The ninth step is reserved to reflect on the discussion, which could resemble the reflection in the scientific method described here. The tenth step, however, which is reflecting on the thinking process, is not integrated into the scientific method. I suggest adding such a step to advance the way students think about and assess their own thought process that has gone into designing the experiment, the conclusion they have drawn, and additional questions they may wish to think about for further research.

#### Virtues-valuing

While respecting the conflicts and debates over the definition of virtue throughout history, exploring these various definitions is not the subject of this article. It is sufficient to adopt the Cambridge Dictionary definition of virtues as good moral quality in a person, or the general quality of being morally good.

Despite the fact that this definition is loaded with terms that need to be defined, our purpose here is to evaluate the virtue-valuing strands in P4C and the scientific method. Moral growth, on the other hand, is understood as the achievement of a full life, including that of becoming a good person [44]

Since P4C is an applied means of philosophy, the students in P4C do not read about philosophical virtues and ethics. Rather, during dialog, they discuss philosophical issues that arise from their own experiences. During the discussion, participants can think for themselves about the essence of the concept of discussion. The P4C process itself contains some philosophical qualities and virtues [45], such as good judgment and reasoning, that are exercised while practicing P4C [46].

The P4C effect on participants’ virtue-valuing was challenged by Franken Figueiredo, who insisted that the founders of P4C believed that it was an appropriate method of moral education that could teach intellectual virtues to the participants. P4C falls short because the founders did not provide an appropriate explanation for how P4C ensures this virtue learning through the program. In other words, if the participants are required to perform intellectual virtues during the extraction of the concepts related to virtues, we can say they may be motivated to apply them. However, there is no guarantee that they will always apply these virtues correctly [47].

Practicing the scientific method requires reasoning and good judgment among other intellectual virtues. Also, for scientists to accept or reject a theory, they have to apply theoretical virtues to examine it. The theoretical virtues make up the trait that shows that the theory is true or worth accepting [48]. Thus, while using the scientific method, students are practicing theoretical virtues, such as consistency, accuracy, simplicity, unifying power, and fertility [48,49]. Members of the scientific community are required to clarify on what basis they have accepted or rejected a scientific claim.

Both the scientific method and P4C value virtues are implemented by applying the process in the real world. Scientists are expected to learn certain qualities throughout the process.

### Trompenaars Hampden-Turner™ dilemma reconciliation model

**Fig. 3:**
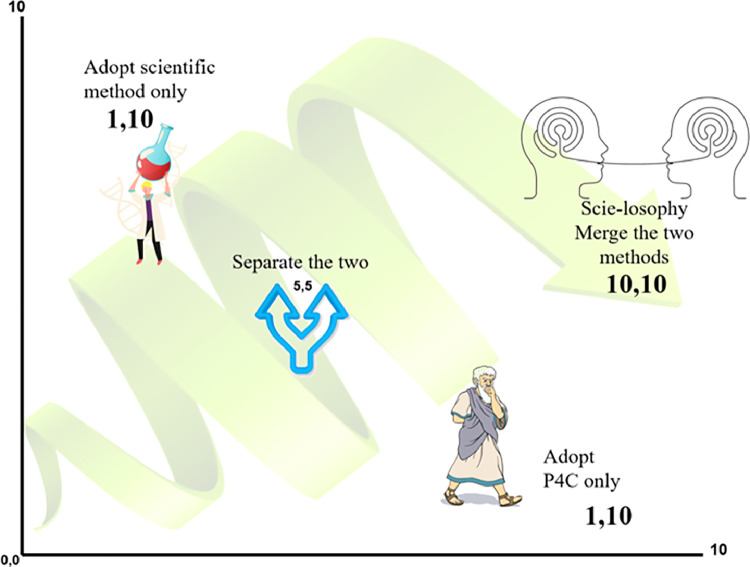
Reconciliation of the P4C versus the scientific method dilemma.

The analysis of P4C and the scientific method using the six strands of effective education reveals a dilemma for educators: how to reconcile the fully open style of P4C with the structured evidence-based approach to education. To address this dilemma, we employ the dilemma reconciliation model, which starts by expressing the situations as dilemmas. Second, these dilemmas are charted and codified in a standard style by placing the scientific method of teaching on one axis and P4C on the other and then providing the model (reconciliations) to these two teaching approaches (Fig. 3). Third, stretching the dilemma: each side is examined for its advantages through the eyes of its supporters, and disadvantages through the eyes of their extreme antagonists from the other party. The purpose of stretching the two parties is to be able to see the epithets. Then to think impartially, we ideally need to start at the (0,0) in Figs. 2 and 3. which is easier in a multicultural background. Then, after the whole picture is obvious to you, you work your way in a continuous learning loop [50], moving from side to side so that movement strengthens the logic of both sides, until you reach a reconciliation state (10,10). In practice, depending on culture and academic background it is expected for the process to commence from one of the sides (1,10) and gradually progress toward the middle ground by considering both sides' perspectives.

#### Stretching the dilemma

The P4C

We present the advantages and disadvantages of P4C in published research and personally collected opinions of P4C facilitators and trained teachers.

Advantages:

1. Improves students’ critical thinking and creative skills [51], [52], [53], [54].
2. Improves students’ focused listening and critical evaluation of information before conclusions [54].
3. Improves students’ abilities to make connections, reflect, build concepts, and provide explanations [53,54].
4. Improve students’ abilities to produce higher-order questions [55].
5. Helps teachers’ effectiveness and professional development through engagement in dialog, practice, and reflection [56].
6. Improves the students’ reading reasoning, and non-cognitive skills [57], [58], [59].
7. Provides a safe environment and increases openness, democracy, and mutual respect among students [53,54,[60], [61], [62]].

Disadvantages:

1. With P4C all you have is opinions and no innovative progression.
2. “There is no evidence that P4C had an impact on children's reading outcomes, math, social and communication skills” [63].
3. Difficulties with pupil engagement [64].
4. Time-consuming and discusses unimportant concepts [60,63,64].
5. Diminishes writing skills. P4C leads to not producing written evidence of pupils’ achievements [64].
6. Increased the vulnerability of students with special cases [61,64].
7. Questionable ethics of developing pupil (during critical developmental stage) expectations counter to prevailing educational practice [64].
8. Unfair and unjust to “employ enquiry in a neutral and an impartial fashion without considering the democratic contexts children encounter” [65].
9. Not as transformative, as revolutionary, or as radical, as it is desirable for it to be to make any difference [65].

The Scientific method

Advantages

1. Increases problem-solving skills [1],
2. Increases critical thinking as it supports empirical evidence, proof, and verification.
3. Increases reasoning and observation skills.
4. Practical and reliable at finding the truth.
5. Increases objectivity and impartiality.
6. Progressive.

Disadvantages

1. “Naive emulation of the scientific method”: Laboratory experiments are performed in cookbook fashion, without understanding the underlying substantive and methodological principles involved [1].
2. Solving problems and pouring reagents are not sufficient to discover all of the important concepts and generalizations they need to know [1].
3. It is pointless to use discovery techniques to develop an intuitive understanding in this developmental stage [1].
4. Not realistic, as available procedures must be skillfully “arranged” for the students to make the final result almost inevitable [1].
5. Time-cost restrictions [1].

#### Working towards reconciling the methods

**Fig. 4:**
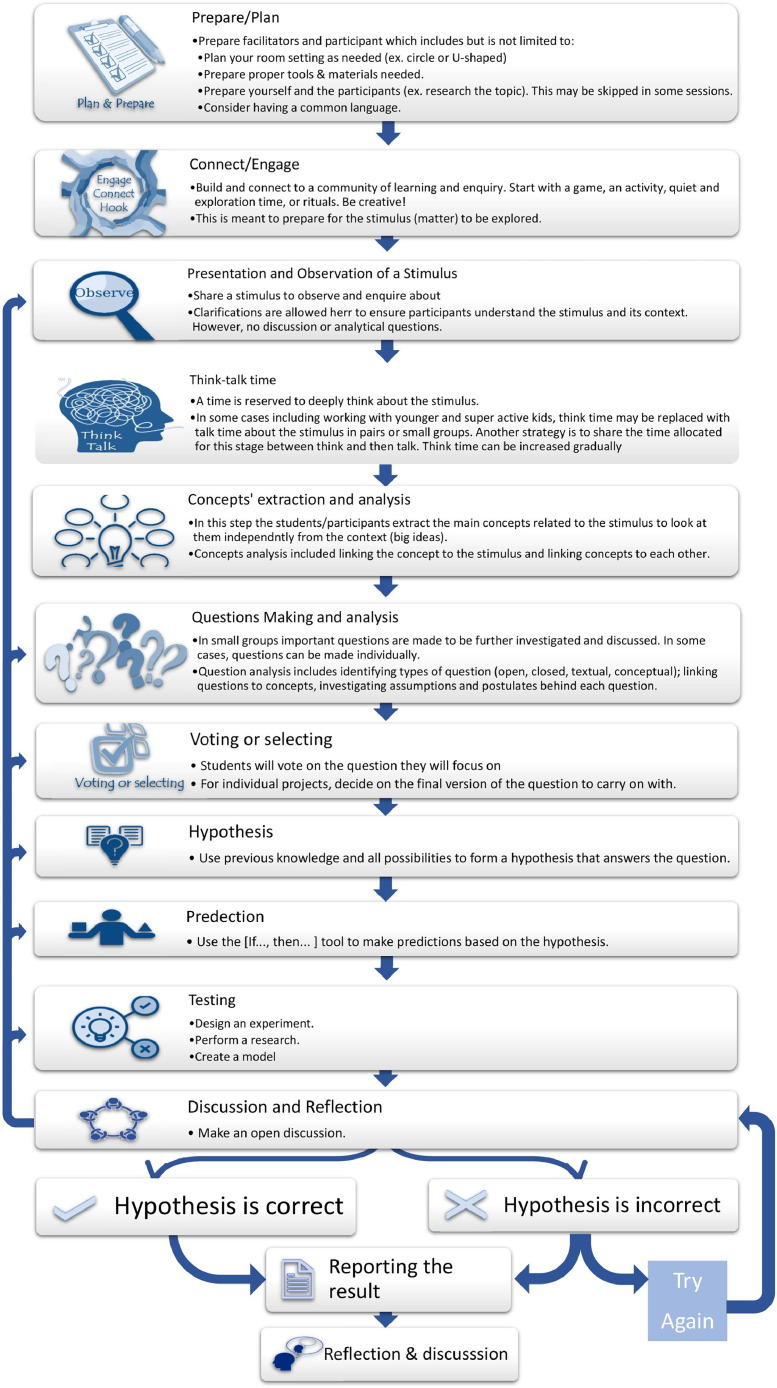
proposed Scie-losophy model.

Ab Wahab (2022) reported a recent systematic review that evaluated the published benefits and challenges of P4C. In their review, they called for future research to overcome the challenges faced by teachers and students when implementing P4C by providing teaching modules using P4C for teachers. For the constructed stimulating module, they required appropriate materials for the students’ cultural environment, religion, and level of thinking [14]. This solution implies that P4C is to continue being used as it is to supplement some lessons which means science classes for instance will continue to use the scientific method. This is considered in the Dilemma theory as the compromise between the two methods (labeled 5,5 in Figs. 2 and 3). Performing both P4C and the scientific method independently does not enable the reconciliation continuous learning loop [50]. Where (10,10) represents the reconciled proposal between the P4C and the Scientific method. The reconciliation of this dilemma, P4C versus the scientific method, is presented in Fig. 4.

### Proposed model

My analysis shows that, while each method offers more than the other regarding some strands, both the scientific method and P4C satisfy the six strands to a certain extent. Adopting one approach above the other will yield less than optimal results. I am strongly discouraged from following a saturated educational system in which one of the two methods is used exclusively. Fig. 4 shows the proposed model “Scie-losophy model for learning and thinking”. This model sees the best of the two words that combine the rigor of scientific methods with more creative, open, and reflective methods resulting in rationally constructivist outcomes.

In the model, the frame word presented in Fig. 4, all steps of the Scie-losophy can be followed in the same order for full sessions or full projects. On the other hand, sessions can include only some of the steps as needed or as time permits.

#### Action plan, implementation, and mentoring

The model proposed in this paper (Fig. 4) is a result of years of facilitating P4C sessions and practicing the scientific method as a student and as a scientist teaching science. We will continue designing modules, applying the proposed method, reflecting, and analyzing results. We also invite interested colleagues and researchers to consider applying the proposed method and share feedback.

## Conclusion

Overall, it is clear to me that, while one method may be superior to the other with respect to some strands, both the scientific method and P4C satisfy the six strands to a certain extent. I believe it is counterproductive to the learning process to propose the adoption of one method over the other. One strand of research is not better than the other. The father of P4C, Matthew Lippman, conducted an experiment in which he supplemented a group of students with eighteen sessions. Each session lasted forty minutes. The sessions were conducted twice a week. In his conclusion, he stated:

*“I am now; convinced that philosophy can and should be a****part****of the entire length of a child's education.”*[4]

He was clear in his proposal that he wanted to foster logic and reasoning in children through philosophical discussion. It is important to note that he did not claim that we should exclude other teaching methodologies. P4C's critique of other methodologies will not help create an inclusive learning environment. On the contrary, it may result in isolating P4C from other successful methodologies instead of supporting them. It is clear that P4C is a method to help students, but not on its own. P4C allows learners to free their minds, speak confidently, articulate their ideas, and allows them to use reason and logic as a tool. As a supporter of P4C as a method for democratizing education, I would refrain from promoting it as the savior of education as implied and stated by P4C supporters [66].

Taking a more moderate approach, I propose in Fig. 4a reconciliation between the scientific method, which is a philosophical branch in its essence, and P4C. In this proposed model, P4C offers more emphasis on pausing to think conceptually and to reflect on various cognitive processes. The scientific method offers the collection of evidence and the practical experimental method to test and draw a conclusion. A few other elements are added to ensure that the newly proposed method reconciles available methods to provide an overall comprehensive approach that can advance and further learners’ acquisition of knowledge. It is important to note that I don't claim that this method, or any method, works for every student and all the time. Teachers need to be flexible.

Several paths are open for future research including designing modules using the Scie-losophy model, evaluating the benefits and limitations of the model practically, and measuring the long-term effect of the proposed model.

## Ethics statements

This research does not involve human subjects, animal experiment, not data collected from social media.

## CRediT authorship contribution statement

**Amal A. Alsufyani:** Conceptualization, Methodology, Formal analysis, Writing – original draft, Writing – review & editing.

## Declaration of Competing Interest

The authors declare that they have no known competing financial interests or personal relationships that could have appeared to influence the work reported in this paper.

## Data Availability

No data was used for the research described in the article.
